# Beyond the horizon: immersive developments for animal ecology research

**DOI:** 10.1186/s42492-023-00138-3

**Published:** 2023-06-20

**Authors:** Ying Zhang, Karsten Klein, Falk Schreiber, Kamran Safi

**Affiliations:** 1 Department of Computer and Information Science, University of Konstanz, Konstanz, 78464 Germany; 2 Department of Migration, Max Planck Institute of Animal Behavior, Radolfzell, 78315 Germany; 3 Faculty of Information Technologies, Monash University, Melbourne, VIC 3145 Australia

**Keywords:** Immersive analytics, Animal ecology, Collaboration, Interactive data visualization

## Abstract

More diverse data on animal ecology are now available. This “data deluge” presents challenges for both biologists and computer scientists; however, it also creates opportunities to improve analysis and answer more holistic research questions. We aim to increase awareness of the current opportunity for interdisciplinary research between animal ecology researchers and computer scientists. Immersive analytics (IA) is an emerging research field in which investigations are performed into how immersive technologies, such as large display walls and virtual reality and augmented reality devices, can be used to improve data analysis, outcomes, and communication. These investigations have the potential to reduce the analysis effort and widen the range of questions that can be addressed. We propose that biologists and computer scientists combine their efforts to lay the foundation for IA in animal ecology research. We discuss the potential and the challenges and outline a path toward a structured approach. We imagine that a joint effort would combine the strengths and expertise of both communities, leading to a well-defined research agenda and design space, practical guidelines, robust and reusable software frameworks, reduced analysis effort, and better comparability of results.

## Introduction

**Fig. 1:**
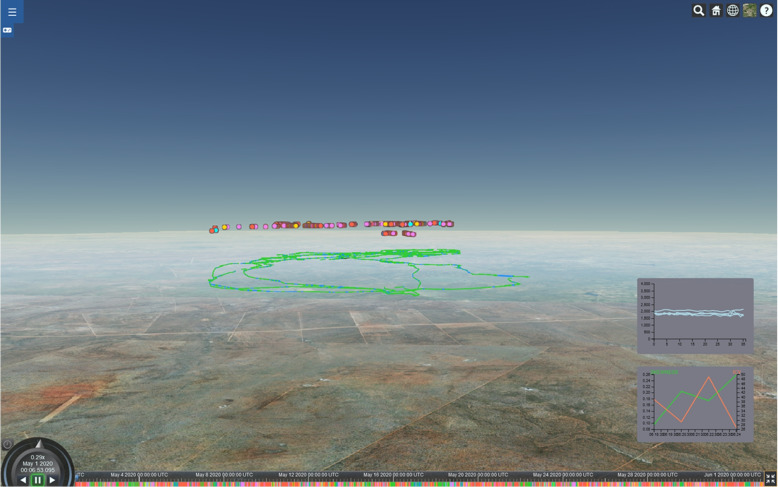
Visual analysis in the TeamWise animal movement analysis tool [15], showing a behavior classification visualization along a movement trajectory on top of satellite imagery. The timeline bar at the bottom shows behavior categories along the time axis, and the behavior annotations are embedded in the 3D view. 2D charts show visualizations of additional abstract data

Rapidly emerging technologies, such as lightweight sensor tags and advanced satellite imagery, provide unprecedented access to large and quickly increasing amounts of data on animal movement and behavior, as well as the corresponding environmental conditions. The sheer volume, scale, and complexity of the data and the associated uncertainty create challenges for analysis and interpretation. These challenges include questions regarding computer-based handling, such as pre-processing, integration, automated analysis and representation, and human interaction with and interpretation of the data. The wealth of data also creates an opportunity by facilitating the investigation of more holistic research questions, considering several aspects of animal behavior simultaneously with environmental conditions. Examples include supporting machine learning [1–6], enriching models of behavior with facets that were previously unresolvable [7–9], and providing a broader base for the interpretation and detection of patterns or traits on a more fine-grained spatial and temporal level [10, 11]. This opportunity is particularly welcome at a time when rapid environmental changes, owing to climate change and human impact, and corresponding changes in behavioral patterns might require adaptive approaches and a revisitation of established models and views [12–14]. Consequently, an increasing number of tools are available that integrate data on animal movement and behavior, as well as information on the movement environment, with analytical methods to create interactive interfaces (Fig. 1).

**Fig. 2:**
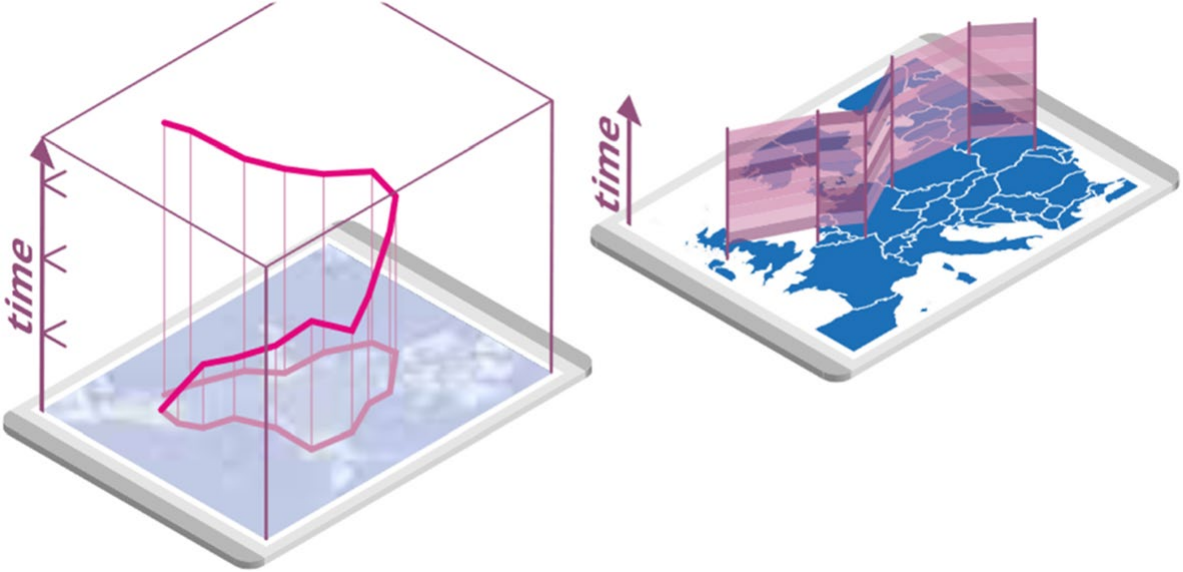
Superimposed visualization concept example for movement data analysis on a map, e.g., using AR visualization aligned with a tablet (taken from ref. [16])

**Fig. 3:**
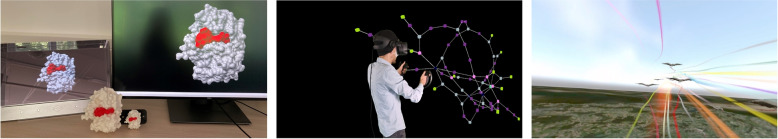
Examples of using immersive technology. Left: Molecular representation on a glass-free 3D Looking Glass device, as a 3D-printed physicalization that can be used for interaction and haptic feedback, positioned next to a standard 2D monitor representation; Center: Interaction with a network visualization in VR; Right: VR view of a flock of storks soaring from a stork’s perspective (taken from ref. [20])

Meanwhile, we have new technologies that can facilitate data analysis. The development of IT technologies, such as virtual reality (VR) and augmented reality (AR) environments, large high-resolution monitor walls and touch surfaces, holographic displays, and interactive 3D visualizations, have the potential to greatly improve the scope and efficiency of animal ecology analysis. Suddenly, we can superimpose data visualizations on maps on the fly using mobile devices [16] (Fig. 2), “fly with the flock” [17] (Fig. 3, right), or recreate virtual environments for the study of animal behavior in a controlled setting [18, 19].

In the endeavor to take advantage of the resulting opportunities, biologists and computer scientists face common challenges. Both seek ways to create scalable and robust solutions [21, 22] for faithful and reliable human interpretation [14, 23]. Similarly, both investigate approaches that exploit new technologies for this purpose.

However, how to best exploit these technologies and integrate them into a human-centered approach has not yet been well defined [18, 21, 24–26]. A joint community effort across both communities could save resources, provide joint software platforms, and significantly improve the quality and acceptance of the proposed standards and results.

Immersive analytics (IA) [20, 27] is an emerging research field in computer science that investigates the potential for immersive technologies to be used to improve data analysis and communication, highlighting the potential to reduce analysis effort, widen the range of questions that can be tackled, and improve outcomes. We propose that biologists and computer scientists combine their efforts to lay the foundation for IAs in animal ecology research. We discuss the potential challenges and outline a path toward a structured approach.

Joint research should define guidelines and standards, highlight best practices, characterize the design space for solutions, and address important challenges and research questions. In addition, reusable software frameworks that reduce implementation effort and facilitate reproducible analysis workflows are a major aim.

## IA for animal ecology

### IA

IA aims to create more engaging and immersive experiences and seamless workflows for data analysis applications [28] by exploiting the affordances of devices and immersive environments (IE), such as user movement tracking, stereoscopic 3D (S3D) [29, 30], multimodal interaction [31, 32], and data physicalization [33]. It is ready to facilitate the analysis of the growing amount and complexity of data in animal ecology research and can provide more efficient and powerful animal ecology analysis tools and environments.

However, to this day, IA is mainly concerned with fundamental research questions rather than practical applications. Consequently, overarching research questions for IA [20, 34, 35] are as follows:

- Use of S3D – how can it be best exploited, which representations are best suited, how does it compare to standard 2D, how to overcome challenges of scale, location, perspective, and depth, and how to integrate classical 2D representations [30, 36]?
- Use of multimodal representation – how can sensory channels be further employed to go beyond the capabilities of visual representation, e.g., through the use of sonification, haptics, or data physicalization [33]?
- Interaction with data representations and the user interface for analysis – how to create efficient and intuitive interfaces, e.g., using multi-modal interaction [37, 38] and transitional interfaces [39, 40]?
- Navigation – what are good navigation metaphors to allow the user to traverse large data sets while maintaining orientation and supporting the generation of a mental map, i.e., the internal representation of knowledge concerned with the data and its connection to external representation in the IE [41]?
- Scalability – how to cope with huge data sets, regarding the computational requirements and responsiveness of automated analysis but also aggregation and abstraction for human interpretation?
- Collaboration – how can collaboration for analysis be explicitly supported, e.g., given the large physical immersive spaces provided by IE [42]?
- Analysis and presentation environments and audience – how to tailor an approach for the constraints of an environment, e.g., lab vs in the field, or a group of users, e.g., experts performing exploratory analysis vs decision makers using communication of results?

Nevertheless, IA has been commended for use in a variety of areas [43–49], (Fig. 3). However, efficient and user-friendly approaches must be tailored to the specific questions and requirements of an application.

The combination of data visualization, multi-modal data representation, multi-modal interaction, integration of analysis methods, and device and environment characteristics constitutes a large design space. Thus, suitable solutions within this space must be designed and evaluated for use in animal ecology research with a focus on specific tasks, users, and data to fully benefit from the capabilities of IE compared to those of classical desktop environments. To this end, proper use has to be made of the differences in environment characteristics, e.g., regarding the physical immersion of the user, S3D, field of view/regard, user movement tracking, gesture recognition, and interaction using hand-held controllers.

**Fig. 4:**
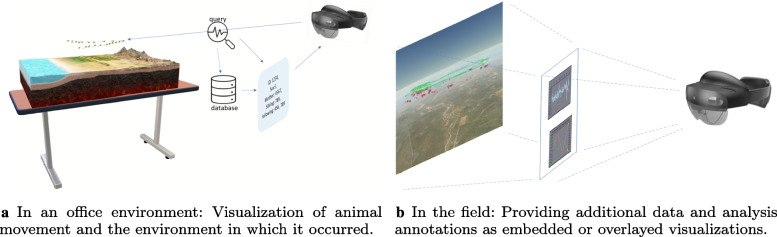
Use of AR technology: Further analysis results and annotations, such as those regarding environmental conditions, can be calculated or interactively retrieved from databases. They can be used as a visual overlay or integrated into the scene. Creating such spatially situated visualizations, i.e., visualizations integrated with a real-world referent such as physical spaces or entities, is a core challenge of IA [50, 51]

A combination of more intuitive interaction with the data, 3D representations for abstract and spatial data [30], and integrated interfaces for automated analysis methods has the potential to greatly improve the analysis cycle, enhancing user experience and efficiency. Possible examples of how this might work for animal ecology research include observing the environment from an animal perspective, walking through a scene of interacting animals while being able to steer an analysis interactively, and combining these scenes with classical abstract data visualizations (Fig. 4). These examples can also be extended by adding support for collaboration, by providing several analysts with the same data representation and allowing shared annotations and analyses, for example. In addition to software frameworks and in cases such as VR HMDs, full software eco-systems, have been developed around immersive hardware technology, significantly reducing the threshold to prototype and develop immersive visualizations as well as integrate automated analysis solutions. Thus, the current situation provides an ideal foundation for investigating the potential of such environments for animal ecology data exploration and analysis.

### Challenges from animal ecology

The relationship between animals and their environment is complex, and animal activity area characteristics depend on the location of suitable living conditions, that is, based on the specific features of an area [52]. Meanwhile, animal behavior can also heavily affect the environment in a variety of ways, such as through pollination, grazing, and the arrival of invasive species. Therefore, current research in animal ecology is also concerned with the identification of features and stimuli that inform animal decisions, trigger actions, influence behavior, and facilitate orientation and navigation. Many analyses, such as those of habitat and corridor configuration, foraging quantity and quality, and migration paths, involve the application of environmental feature data.

The important research questions are often interrelated:

- Investigation of movement and movement patterns on different levels of scale [53–55] and individual or collective movement [24], such as home-range, territorial behavior, and swarm movement
- Interaction between individuals or groups [56, 57], e.g., in movement, predation, and decision making [58]
- Impact of environmental conditions, e.g., on decision making, social dynamics, or survival [18, 19, 59, 60]
- Differences in species and groups, e.g., based on phenotypic variation [61] or related to evolutionary relation
- Cognitive processes underlying behavioral patterns, e.g., foraging or mate choice [62]
- Prediction and modeling of behavior [13, 63]

The available information on animal behavior and movement is usually collected by sensors, imaging, and subsequent processing of the results. Analysis methods need to exhibit a certain level of robustness toward incomplete and noisy data and be capable of coping with the uncertainty associated with it. Representations of the results must convey the corresponding restrictions and limits of confidence to analysts.

Further, behavior information usually needs to be embedded into the environment in which it occurs to allow proper interpretation [53, 64]. This combination is often challenging because of the sparsity and quality of the data available for both the animals under investigation and the corresponding environment [65]. The data only represent a part of the animal environment, and which type of information extracted from the data best facilitates modeling of the interrelations between an animal and its surroundings and what confidence level can be reached need to be investigated [66–68]. For the design of an analytical concept, methods for the extraction and visual representation of the necessary information need to be conceived. Thus, to analyze animal behavior properly, environmental features must be extracted from the available information, integrated with the automated analysis, and presented in tuitively for interactive exploration by the analyst. Hence, which features can be collected and how they can be provided and integrated into approaches and tools for animal ecology researchers need to be examined.

Given the 3D nature of the animal environment, a representation in S3D is appropriate, and there are indicators that S3D representations have advantages over 2D representations for a variety of tasks [29, 30, 69]. However, such a representation comes with a number of caveats. While the investigation can benefit from the depiction of the natural environment, for example, to create hypotheses for landmarks used for decision-making, the necessary fidelity of the representation is a parameter for IE design. How can the environment be reconstructed for human analysis, particularly when no first-hand experience of local conditions is available? To this end, the integration of imaging methods and subsequent processing for the identification of features is required, and models must be employed to represent and simulate the environment and its features in an IE. Furthermore, whereas interaction with representations in 3D can be designed in a more intuitive manner than in a desktop setup [32], adding interfaces for analysis methods in this setting is a challenge. This includes interfaces for settings and selection, as well as for a well-interpretable representation of intermediate results.

Whereas big data approaches can foster the understanding of the ecology of animal movement and behavior [57, 70], they also provide use cases with specific challenges and requirements for analysis approaches. These include the ability to monitor and analyze large-scale data and to derive patterns and unusual behavior at different levels of scale. One example of such a challenge is the recently introduced concept of “Internet on Animals,” which proposes fine-scale biologging through the combination of WiFi and multi-sensor devices. The proposed architecture supports big data biologging, particularly the collection of movement and locomotion data, over extended periods of time. Thus, the analysis environments for such data must be able to handle the scale of the incoming data. Applications might require real-time monitoring as well as aggregation and abstraction of long-term data for trend and pattern detection, along with comparison, for example, between animals or time periods. Important features of the data required for the analysis must be preserved in the aggregation process and made available to the analyst, either in an overview or on demand [70, 71]. The ability to integrate different levels of scale, for example, from different data collection technologies and sampling strategies [70, 72], poses another challenge. This requires proper handling, representation, and navigation techniques. An example would be smooth transitions between different levels of temporal or spatial scales. Further challenges include surveillance for the monitoring and prediction of environmental changes or events. Interesting use cases include the monitoring of the impact of human land use and climate change and the attempt to predict natural disasters by detecting unusual animal behavior [73].

### Potential for synergies and research

IEs have applications in animal ecology education [38], outreach, decision making, and the investigation of behavior. In a holistic approach, the enriched recreation of the environment and information on animal behavior can be combined with access to analysis methods and pipelines, supporting an immersive interactive experience and analysis. For example, Harel et al. [59] discussed the representation of arboreal animal movements and decision making in VR. They mapped the 2.5D setting of canopy environments into a S3D environment for detailed analysis. As the ability to precisely measure movement and position is improving, more fine-grained options for representation are becoming available, which may support a better understanding of animal decision-making, for example, regarding the trade-off between risk and reward [59]. However, as Harel et al. mention, current technologies used to collect data, such as sensor collars or drones, may still have an impact on the available decision options of the animal and the decisions taken.

IEs can provide further advantages for the analysis of such environments by supporting spatial sound representations of collected or simulated data that foster further insight into environmental characteristics, decision drivers, and variations across taxa [74, 75]. Klein et al. [17] performed a benchmark study to assess the suitability of different IEs for animal movement visualization from both the developer and analyst perspectives. They concluded that suitability is strongly dependent on the specific environment and design in relation to the task. Examples include analysis in the field or the lab, collaborative analysis, and decision-making and engagement of the general public.

The use of IEs is not restricted to the representation and analysis of data but can also be used to create environments for controlled studies. Design guidelines and experimental evidence from IA may help to improve environmental design. Sawyer and Gleeson [76] summarized the use of VR for animal behavior investigations in biological laboratories. Their summary includes neurological studies that involved the placement of animals in a VR environment for controlled behavior studies, as well as studies that promoted the investigation of virtual animal models to replace traditional animal models in biological laboratories. Corresponding results might also inform studies on human-animal interactions, for example, a review on human-dog interactions in VR and AR presented by Oxley et al. [77]. Computer-mediated visual stimulation of animals for behavioral research has been employed for many years, and VR was successfully adopted several years ago. For instance, see the survey on VR systems for rodents presented by Thurley and Ayaz [78]. In this study, they advocate for the transfer of concepts tackling research questions of spatial cognition and navigation from human to animal behavior research. However, Thurley and Ayay also discussed the issue of the trade-off between stimulus control and restraint, which is greater for animals as they cannot be instructed before a study. Restraints, such as movement fixation, might restrict movement options, limit necessary sensory input, or even lead to unintended deviations from real-world experiences, including conflicting sensory information, which might be a confounding factor in the investigation. Taube et al. [79] discussed the problem of using results from virtual setups to interpret spatial orientation and navigation, as these setups do not factor the activation of motor, vestibular, and proprioceptive systems. To overcome these restrictions, Stowers et al. [80] presented a VR system for freely moving animals. Naik et al. [18] presented a review of animal behavior experiments conducted in virtual environments and argued that while virtual environments have become a widely used tool for animal behavior research, more interdisciplinary research is required.

Open research questions in IA for animal ecology include how to integrate data on animals and the environment into automated analysis, which IE is best suited for a specific analysis task, how to best represent data and analysis results within the IE, and how to support the exploration of the data through intuitive interaction and navigation approaches (see Toward a structured approach for animal ecology IA section). Only initial investigations from the computer science field have targeted aspects of animal ecology, particularly regarding geo-visualizations. Examples include the use of globes and maps [81] and differences in IEs for bird movement analysis [17].

Given the opportunities of IEs, including not only the larger visualization space and up to six degrees of freedom for the analyst’s movement in the data representation, but also more intuitive interaction, several advantages for animal ecology are envisioned. First, the 3D visualization space can be used to better show animal behavior in the environment in which it occurred (Fig. 4a). Next, the analyst can be immersed in the scene if necessary, exploring different perspectives, for example, from an animal’s viewpoint. In addition, interaction with the scene can be more direct; an example would be selecting animal representations by hand for further analysis. Finally, the integration of automated analysis into scene depiction makes the switch between result representation, data exploration, and interaction with analysis methods unnecessary.

However, the value of such solutions depends on how they support the complex analysis of animal behavior and the interplay with the environment, how they can be integrated into everyday analysis work, and how they can intuitively be used by domain experts. Analysis aspects, such as interactions between animals, movement patterns, influential environment characteristics, and drivers of animal decisions, must be integrated in the analysis and properly represented for human reasoning. We assume that different use cases will benefit quite differently from affordances of IEs and, thus, will need specific design solutions.

**Fig. 5:**
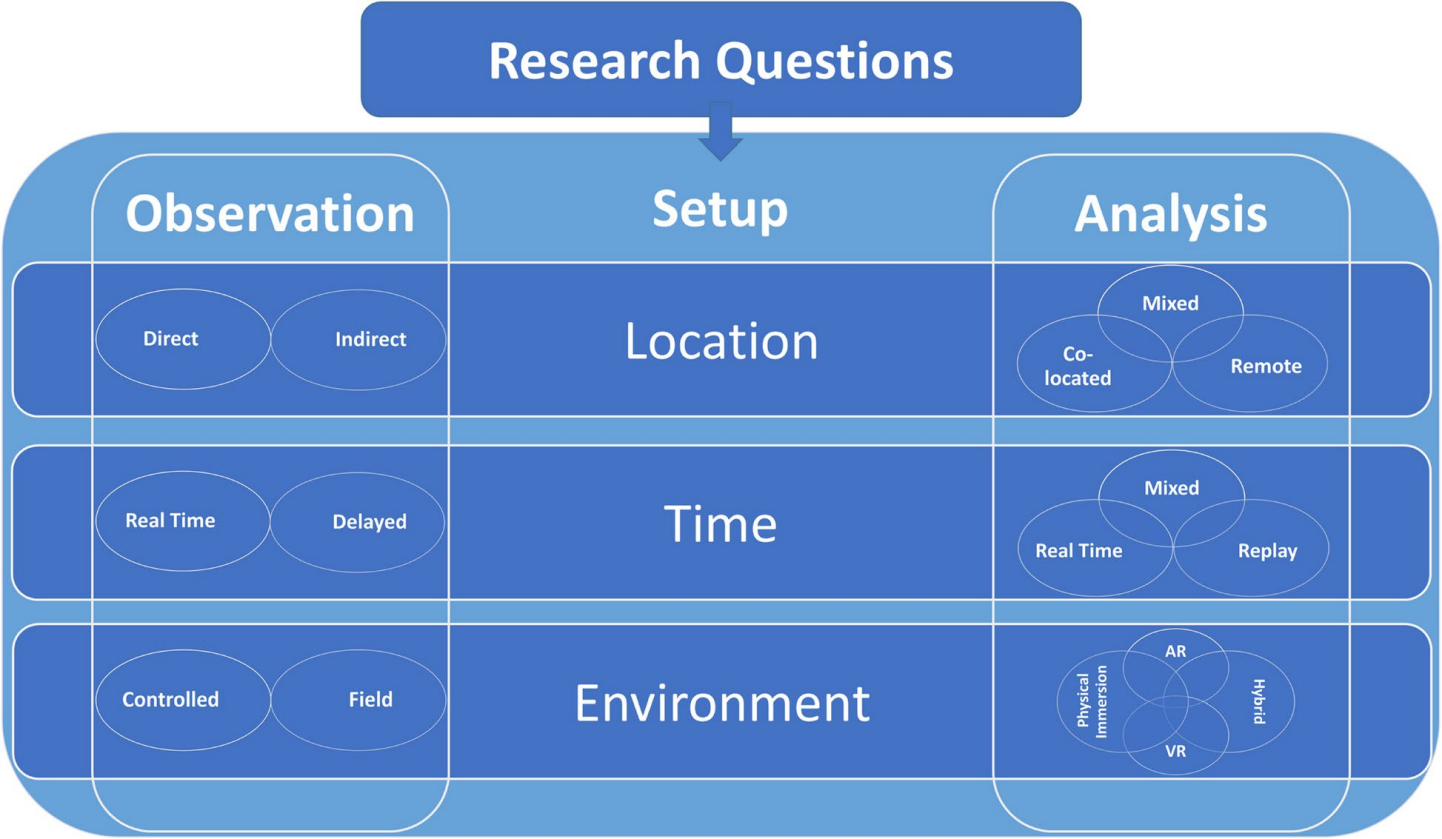
The aspects that distinguish use-cases for application of IA in animal ecology cover several major categories related to location, time, and environment

The main categories that distinguish the use cases include the spatio-temporal and environment conditions of the observation and analysis (Fig. 5). The observation can be

- direct, i.e., an analyst observes the animal through the senses and also perceives the context of the surrounding environment, or indirect, in which sensors are used to observe a selected set of certain features and parameters of animal behavior and environment;
- in real-time, for instance, when an observer is in the field simultaneously with the animals under observation, or asynchronously, i.e., with a delay between the animals’ action and the observation of its results or remainders (e.g., tree marking or nest construction);
- in the field or in a controlled environment, for example, with a specific experimental setup in a confined lab space to test a hypothesis.

In particular, for direct observations in real time, the observer might also be the analyst or prepare information for later use by an analyst.

The analysis can be

- collocated, i.e., in the same space as the animals, or remote, for example, in the analyst’s office or data theater,
- in real-time, i.e., while observing animals, or asynchronously, for example, by using a replay of the behavior or investigating collected data at a later point in time,
- in a purely physical environment, or computer-mediated environment, for example, fully computer-mediated VR representations of animal behavior in an environment, AR superimposed visualizations, or a hybrid setting, e.g., by projecting an overlay of the environment in which the behavior occurs on an office desk (Fig. 4a),
- in the field or in a controlled environment similar to that of the observation.

To foster the active development of corresponding solutions, a requirement analysis and an exploration of concepts, methods, and designs are required. These steps should be performed in a joint effort by biologists and computer scientists. Thus, research could greatly benefit from focused collaboration between both groups to define a design space for solutions.

To properly assess possible designs for IE, we can first analyze the use case at hand and its requirements regarding the above categories, resulting in a combination of features such as direct and real-time observation in a controlled environment with asynchronous analysis in a VR environment. Afterwards, fitting designs and technology options can be chosen to support the analysis, as well as the observation, for example, by a data overlay through AR in the field.

Some of the resulting possible combinations are more promising than others, and for some, the potential benefits are unclear. For example, the use of VR in a direct observation setting seems artificial because the use of VR hinders direct observation. However, in a collaborative setting with multiple analysts, of which only one uses VR, the case might still be justified. However, in general, we assume that an AR setting is more suitable for direct observation and collocated analysis. Note that mixed scenarios of these categories are also possible, for example, in such a collaborative setting in which roles in a team are distributed.

Another important question for IA research and system development is how to measure success, considering the often complex workflows and long-term cyclic processes that are targeted.

## Interdisciplinary collaboration

While there are already ongoing and successful collaborations between computer scientists and animal ecologists, the current state of research offers the opportunity to shape the research direction of IA for animal ecology by laying the foundation for a community effort. In this effort, the communities can work together to identify the main challenges and coordinate work on common standards and platforms, fostering better exchange, reuse, and comparison of approaches. There are large differences in methodology, vocabulary, and approaches between the domains of biology and computer science that need to be bridged [82]. Computer scientists are concerned with research questions regarding the methodology and concepts of computational approaches to create effective and efficient methods for data analysis. A large part of today’s work in animal ecology, particularly regarding data processing and analysis, involves computer technology, and many animal ecologists are also early adopters of new technology [21]. While often proficient in practical programming, they need to use such methods to tackle their research questions and improve reproducibility and replicability of studies [83]. However, they often have to deviate from their research focus on computer science, and large efforts have contributed to ad-hoc solutions that are often not reused. Rather than having ecologists spend a considerable portion of their time learning and re-implementing advanced computer science concepts, we suggest developing a unifying framework that targets typical use cases and provides guidance, and establishing implementations of best practice approaches. Therefore, we advocate a structured approach to lay the foundation for developments that exploit the skills and expertise of both sides. Initial steps in this direction have been taken [21, 55, 65].

Instead of selecting existing standard tools, with all their restrictions, animal ecologists and computer scientists together can develop tools and software tailored toward the specific requirements of animal ecology. Such an interdisciplinary collaboration would also allow large-scale efforts to be targeted, such as a common platform for animal ecology IA, which decreases the implementation effort for biologists, speeds up the development process, leads to better-designed analysis environments and well-characterized analysis workflows, and increases analysis efficiency and reproducibility. Interactive interfaces that support the creation and execution of individual analysis workflows in a programming-free manner could decrease analyst effort and improve analysis design, as shown by MoveApps [84] or the Orange [85] data-mining platform.

Success stories for structured interdisciplinary approaches to data analysis exist in other areas, where large interdisciplinary teams define ontologies and standards and frameworks and software libraries provide well-designed analysis tools. Examples include the COMBINE initiative [86, 87], which coordinates the development of community standards and formats for computational models in systems biology, and the Bioconductor framework [88], which provides tools for the analysis of high-throughput genomic data, showing how coordinated long-term efforts can provide interoperable standards, foster exchange, avoid duplicate work, improve the quality of available software and results, and facilitate high-quality communication of said results.

## Toward a structured approach for animal ecology IA

To initiate a larger initiative, a community effort should be undertaken to perform a requirement analysis that characterizes the available and expected data, analysis operations, and workflows in a structured manner. We suggest the formulation of guidelines and creation of frameworks for the collection, storage, and processing of high-quality data to improve the data analysis. Existing concepts from animal ecology research can be extended and improved with the results of computer science research, such as computational modeling, visual representation, and computational efficiency. For example, the variety of pipeline and workflow models can be extended by aspects that have great potential to improve reasoning and decision-making, such as the way analysts interact with the data, encoding used for data representation, and environment within which the analysis is performed.

**Fig. 6:**
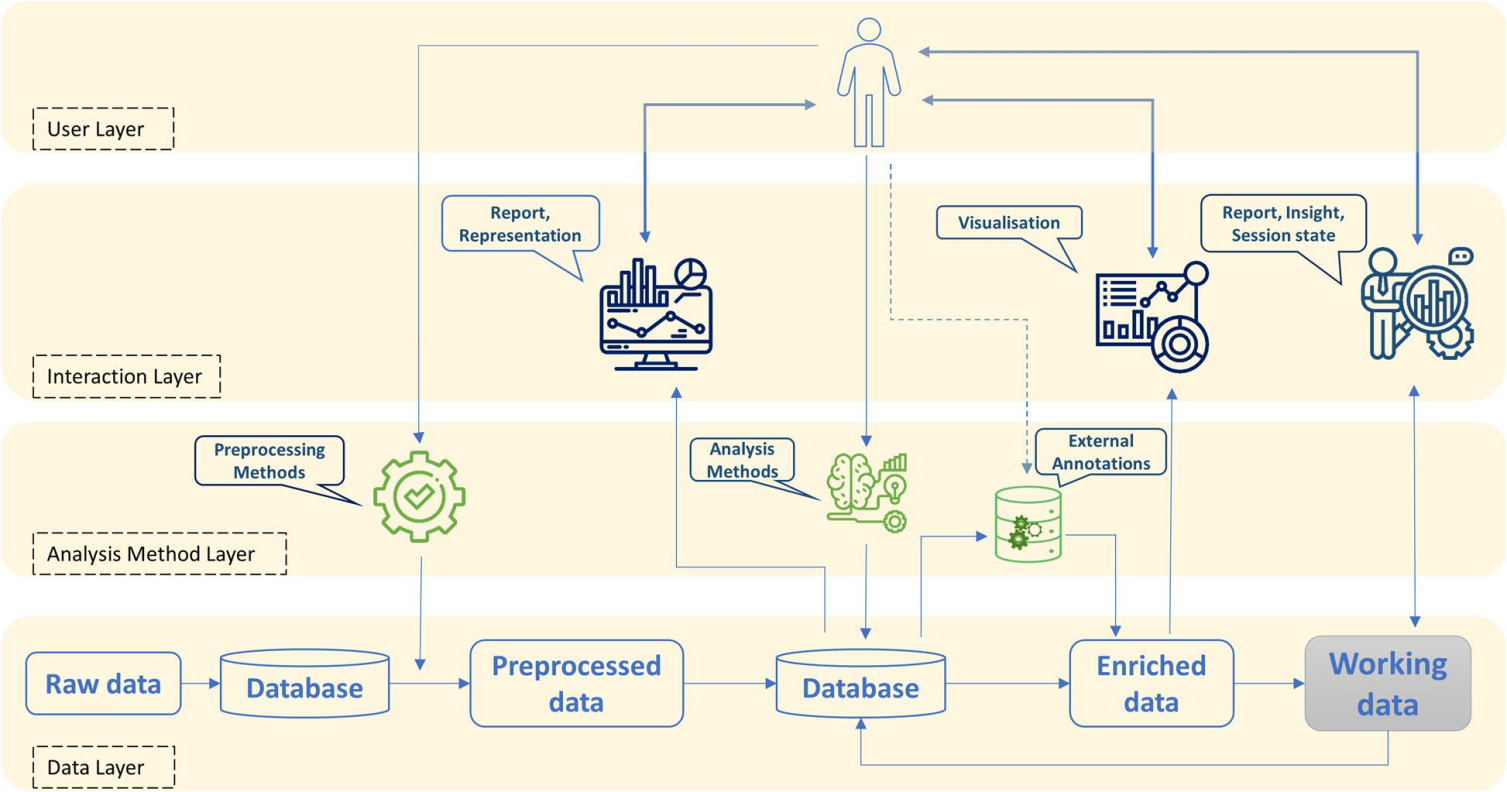
Flow chart of data processing and interactive analysis for animal ecology

Conversely, computer science methods should be extended to address semantics in animal ecology. Many concepts for data modeling, analysis, and representation that have been developed in computer science might be used as the starting point for solutions tailored towards animal behavior research, for example, by integrating semantics and classifications. Thus, standard pipelines and workflows (Fig. 6) should be reinvestigated, enriched, and refined to focus on the specifics of animal ecology. The sensemaking loop model for intelligence analysts [89] is a great example of a refined model that considers the concepts and terminology of the application area. Moreover, it models the process in terms of several cyclic processes, in contrast to basic pipeline models, which often miss the cyclic aspect of data analysis. Motivated by such examples, we propose to characterize the specifics of animal ecology to provide a design space for practical solutions tailored toward IA for animal ecology.

Important aspects that should be considered are data pre-processing, provenance, analysis of spatial-temporal data, collaboration, rendering of a 3D environment, human-computer interaction, environment, encoding, notations, and standards for representation, reporting, and exchange [90]. Whereas such aspects are common in computer science, their combination in the context of decision-making and living organisms is unique.

In the following, we discuss the selected aspects that should be tailored for analysis in animal ecology.

- Data integration and pre-processing: The amount and complexity of incoming raw data is a challenge for analysts and established methods [14, 72, 91]. Different types of data such as data on movement, animal physiology, and the surrounding environment are collected as time series, images, videos, scalar fields, and point clouds. Combinations of such data are required for efficient and effective automated and human analysis [92], and well-specified procedures for the handling of missing data, outliers, inconsistencies, and uncertainty need to be performed. To improve both types of analysis, current approaches need to be adjusted with measures to test/support the data quality, such as automated preprocessing, storage requirements in databases, annotations on the applied processing, mapping of data from different sources, aggregation, specified formats, and standards and ontologies to structure the data. While this challenge has already been tackled for standard environments, some IEs exhibit different limits of scalability and technical restrictions regarding the integration of data pipelines. Moreover, when data representations are designed, information on the preprocessing steps might be required for the analyst to avoid misinterpretation, and robust approaches are required that can cope with quality issues in the data [71]. The integration of data from multiple levels of organization can be used to improve the accuracy of the subsequent analysis [72]; however, careful design is needed, not just for the integration, but also for the corresponding user interface.
- Automated analysis: Given the rich set of already available analysis tools, for instance, provided as R packages, python packages, and machine learning-based methods, solutions for IEs should take advantage of those tools, and concepts are required to provide interfaces that allow seamless integration. This includes not only providing computational access but also considering how a user interface needs to be designed to support a smooth workflow, for example, allowing parameterization of methods. Furthermore, how should the established ways of representing results, such as traditional 2D charts and plots, be transferred into IEs needs to be investigated. As a side effect, such an integration would ease the load when analysts switch between different environments, for instance, in transitional user interfaces [40] where different types of mixed-reality are available to the analyst.
- Cyclic analysis workflows and provenance: The analysis process might be based on an already pre-processed and well-defined set of data that is used for a series of different research questions. For each of these questions, a researcher might revisit the data to explore different aspects. Thus, solutions that make the history of previous investigations available and allow the storage of annotations and partial results are required. In practice, often a cyclic approach is taken [89, 93], in which previous analysis results and additional data can be fed into the investigation. Knowledge generation models focused on animal ecology might help shape workflow design.
- Visual data representation: Available data and technologies facilitate new visual metaphors that might improve the quality and efficiency of the data analysis [94]. However, the potential for animal ecology has not yet been well investigated. For example, whereas publications advocate for the representation of animal movement in the context of the environment in which it takes place [17, 90, 91], the actual impact and optimal ways to do this are not yet clear. For instance, does 3D help or rather distract from the task at hand? Is environment imagery helpful if it does not reflect the situation at the exact observation timepoint? The use of graphical notations to facilitate interpretation and improved comparability is established in many research areas, such as software engineering (unified modeling language) or systems biology (systems biology graphical notation) [94]. Similar efforts could be undertaken in animal ecology to improve analysis and communication and support replicable and well-defined results. With more data sources and dimensions available, proper use of visual variables for data mapping in different IEs, as well as the extension to multi-modal representation, for example, the inclusion of haptics or sound, should be investigated. The visualization of data quality, regarding missing data or uncertainty in measurements, is a further aspect that needs to be considered. When data representations are optimized for IEs, for instance, the use of S3D that allows the user to walk in the representation, the questions of how to share such representations and how to communicate the findings arise. While established charts could simply be printed or shared as images, this might differ greatly for 3D representations.
- IEs: As we have discussed in IA for animal ecology section, the selection of fitting IE designs would strongly depend on data characteristics, research and analysis workflow, and the specific research question under investigation. Which technologies to use and how to employ them needs to be investigated for specific settings in animal ecology. For example, the different classes of animals, available data sources, nature of the investigation, e.g., exploration or hypothesis testing, and the specific task, e.g., investigation of use of resources or group interaction, should be considered. Consequently, we would expect guidelines on what design is better suited for which type of analysis, going beyond the few results for specific classes of animals and environments [17, 81].
- Interaction: We believe that new technologies allow the creation of more engaging interfaces but also require further efforts to design interaction metaphors that support more intuitive, effective, and efficient data analysis and reasoning by animal ecologists. One example is to show the data in a representation of its environment, for instance, in a table-top or room-sized representation, with navigation by movement or gesture, or adaptive representations, facilitating the manipulation of data representations and interface elements in a more natural way. However, the efficiency of interactions and the required amount of user guidance needs to be investigated.
- Collaboration: Collaboration should be specifically integrated in practical approach for animal ecology [95]. This includes data annotation (classifications, personal comments), recording and reproduction of processing steps (provenance) for synchronous and asynchronous collaboration, and methods to support collaborative analysis in real or virtual spaces, for example, through the representation of collaborators to facilitate communication and exchange.

## Action items

The following steps foster a better mutual understanding and collaboration between the animal and computer sciences. While they constitute research in their own right, they can serve as a preparation for further research on both foundational and practical solutions for animal ecology research.

**Fig. 7:**
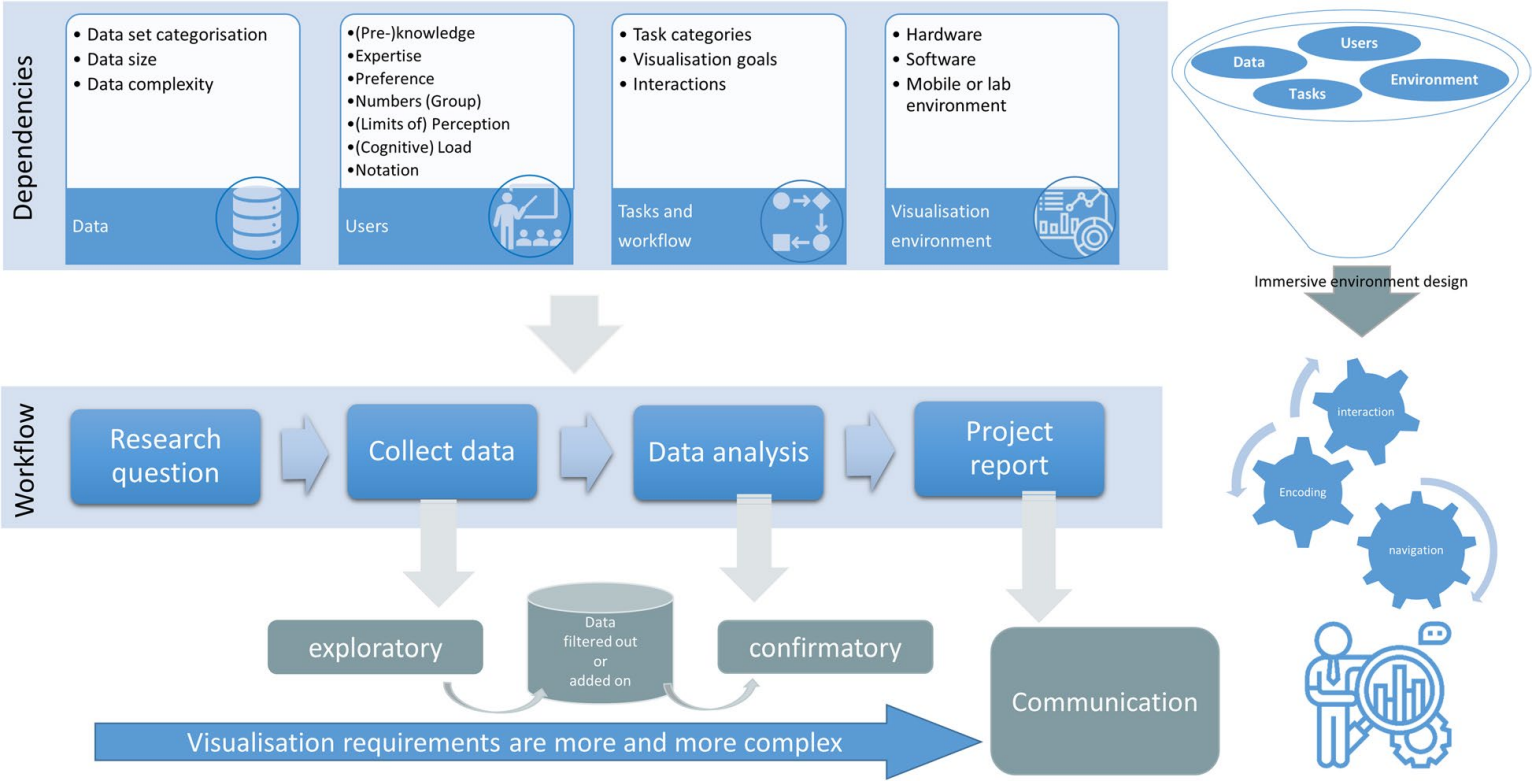
Main aspects of the requirement analysis, including dependencies on data, user, task, and environment, as well as existing approaches and workflows of practitioners

To exploit the potential of new immersive developments in animal ecology research and lay the foundation for unified handling and reproducible results we propose the following:

- Conduct a requirement analysis that covers a broad range of use cases, discussing characteristics of data, tasks, workflows, user roles, research questions, and potential analysis environments. The different aspects of such an analysis are summarized in Fig. 7.
- Evolve existing guidelines and pipelines for data processing and interactive representation or create new ones that fit better the requirements of practitioners in animal ecology research regarding the above listed characteristics.
- Have a close collaboration of biologists and computer scientists to drive the exploration of the design space in the right direction—developments have to be informed by the experience and needs of the practitioners. Thus, common venues should be organized to foster exchange and plan the path ahead.
- Create software support by developing a unified framework that allows for easier implementations, better reproducibility, and comparison of results, as well as a unified user experience. Design considerations based on the requirement analysis can inform a framework architecture in line with analysis workflows.
- Motivate the development and use of notations and standards for storage, exchange, automated analysis, and visual representation.

## Conclusions

We see large potential for the use of IA approaches for animal ecology research as well as in the application of concepts such as standardized notations. However, we think that possible avenues are underexplored and research could greatly profit from a structured collaboration between the animal ecology and computer science fields in the topic of IA. This would allow the enrichment of models and concepts from computer science with the requirements of animal behavior research and shape reusable and durable solutions, such as a unified framework, to exploit the new immersive technologies for data analysis. The first steps could include workshops to foster the exchange and define the challenges and a roadmap, followed by specifications of guidelines and standards, for which interfaces and software platforms can be created.

## Data Availability

Not applicable.

## Abbreviations

VR: Virtual reality
AR: Augmented reality
IA: Immersive analytics
IE: Immersive environment
S3D: Stereoscopic 3D
HMD: Head-mounted display

## References

[1] Valletta JJ, Torney C, Kings M, Thornton A, Madden J (2017) Applications of machine learning in animal behaviour studies. Anim Behav 124:203-220. 10.1016/j.anbehav.2016.12.005

[2] Humphries G, Magness DR, Huettmann F (2018) Machine learning for ecology and sustainable natural resource management. Springer, Cham. 10.1007/978-3-319-96978-7

[3] Angelov PP, Soares EA, Jiang R, Arnold NI, Atkinson PM (2021) Explainable artificial intelligence: an analytical review. WIREs Data Mining Knowl Discov 11(5):e1424. 10.1002/widm.1424

[4] Vilone G, Longo L (2020) Explainable artificial intelligence: a systematic review. arXiv preprint arXiv: 2006.00093.

[5] Linardatos P, Papastefanopoulos V, Kotsiantis S (2021) Explainable AI: a review of machine learning interpretability methods. Entropy 23(1):18. 10.3390/e23010018 10.3390/e23010018 PMC7824368 33375658

[6] Schlegel U, Oelke D, Keim DA, El-Assady M (2020) An Empirical Study of Explainable AI Techniques on Deep Learning Models For Time Series Tasks. arXiv preprint arXiv: 2012.04344. 10.48550/arXiv.2012.04344

[7] Baglioni M, Fernandes de Macêdo JA, Renso C, Trasarti R, Wachowicz M (2009) Towards semantic interpretation of movement behavior. In: Sester M, Bernard L, Paelke V (eds) Advances in GIScience. Proceedings of the 12th AGILE conference, Hannover, June 2009. Lecture notes in geoinformation and cartography. Springer, Heidelberg, pp 271-288. 10.1007/978-3-642-00318-9_14

[8] Wittemyer G, Northrup JM, Bastille-Rousseau G (2019) Behavioural valuation of landscapes using movement data. Philos Trans R Soc Lond B Biol Sci 374(1781):20180046. 10.1098/rstb.2018.0046 10.1098/rstb.2018.0046 PMC6710572 31352884

[9] Joo R, Boone ME, Clay TA, Patrick SC, Clusella-Trullas S, Basille M (2020) Navigating through the R packages for movement. J Anim Ecol 89(1):248-267. 10.1111/1365-2656.13116 10.1111/1365-2656.13116 31587257

[10] Seidel DP, Dougherty E, Carlson C, Getz WM (2018) Ecological metrics and methods for gps movement data. International Journal of Geographical Information Science 32(11):2272–2293. 10.1080/13658816.2018.1498097 10.1080/13658816.2018.1498097 PMC6322554 30631244

[11] Miller HJ, Dodge S, Miller J, Bohrer G (2019) Towards an integrated science of movement: converging research on animal movement ecology and human mobility science. Int J Geogr Inf Sci 33(5):855-876. 10.1080/13658816.2018.1564317 10.1080/13658816.2018.1564317 PMC7531019 33013182

[12] Rotics S, Turjeman S, Kaatz M, Resheff YS, Zurell D, Sapir N et al (2017) Wintering in Europe instead of Africa enhances juvenile survival in a long-distance migrant. Anim Behav 126:79-88. 10.1016/j.anbehav.2017.01.016

[13] Oloo F, Safi K, Aryal J (2018) Predicting migratory corridors of white storks, Ciconia ciconia, to enhance sustainable wind energy planning: a data-driven agent-based model. Sustainability 10(5):1470. 10.3390/su10051470

[14] Abrahms B, Aikens EO, Armstrong JB, Deacy WW, Kauffman MJ, Merkle JA (2021) Emerging perspectives on resource tracking and animal movement ecology. Trends Ecol Evol 36(4):308-320. 10.1016/j.tree.2020.10.018 10.1016/j.tree.2020.10.018 33229137

[15] Klein K, Aichem M, Zhang Y, Erk S, Sommer B, Schreiber F (2021) TEAMwISE: synchronised immersive environments for exploration and analysis of animal behaviour. J Vis 24(4):845-859. 10.1007/s12650-021-00746-2

[16] Langner R, Satkowski M, Büschel W, Dachselt R (2021) MARVIS: combining mobile devices and augmented reality for visual data analysis. In: Proceedings of the 2021 CHI conference on human factors in computing systems, ACM, Yokohama, 8-13 May 2021. 10.1145/3411764.3445593

[17] Klein K, Sommer B, Nim HT, Flack A, Safi K, Nagy M et al (2019) Fly with the flock: immersive solutions for animal movement visualization and analytics. J R Soc Interface 16(153):20180794. 10.1098/rsif.2018.0794 10.1098/rsif.2018.0794 PMC6505562 30940026

[18] Naik H, Bastien R, Navab N, Couzin ID (2020) Animals in virtual environments. IEEE Trans Vis Comput Graph 26(5):2073-2083. 10.1109/TVCG.2020.2973063 10.1109/TVCG.2020.2973063 32070970

[19] Nourizonoz A, Zimmermann R, Ho CLA, Pellat S, Ormen Y, Prévost-Solié C et al (2020) EthoLoop: automated closed-loop neuroethology in naturalistic environments. Nat Methods 17(10):1052-1059. 10.1038/s41592-020-0961-2 10.1038/s41592-020-0961-2 32994566

[20] Klein K, Sedlmair M, Schreiber F (2022) Immersive analytics: an overview. Inf Technol 64(4-5):155-168. 10.1515/itit-2022-0037

[21] Lewis KP, Vander Wal E, Fifield DA (2018) Wildlife biology, big data, and reproducible research. Wildl Soc Bull 42(1):172-179. 10.1002/wsb.847

[22] Shade A, Teal TK (2015) Computing workflows for biologists: a roadmap. PLoS Biol 13(11):e1002303. 10.1371/journal.pbio.1002303 10.1371/journal.pbio.1002303 PMC4658184 26600012

[23] Williams HJ, Taylor LA, Benhamou S, Bijleveld AI, Clay TA, de Grissac S et al (2020) Optimizing the use of biologgers for movement ecology research. J Anim Ecol 89(1):186-206. 10.1111/1365-2656.13094 10.1111/1365-2656.13094 PMC7041970 31424571

[24] Hughey LF, Hein AM, Strandburg-Peshkin A, Jensen FH (2018) Challenges and solutions for studying collective animal behaviour in the wild. Philos Trans R Soc Lond B Biol Sci 373(1746):20170005. 10.1098/rstb.2017.0005 10.1098/rstb.2017.0005 PMC5882975 29581390

[25] Billington J, Webster RJ, Sherratt TN, Wilkie RM, Hassall C (2020) The (under) use of eye-tracking in evolutionary ecology. Trends Ecol Evol 35(6):495-502. 10.1016/j.tree.2020.01.003 10.1016/j.tree.2020.01.003 32396816

[26] HerdHover (2020) HerdHover project webpage. https://herdhover.com/. Accessed 17 May 2023

[27] Dwyer T, Marriott K, Isenberg T, Klein K, Riche N, Schreiber F et al (2018) Immersive analytics: an introduction. In: Marriott K, Schreiber F, Dwyer T, Klein K, Riche NH, Itoh T et al (eds) Immersive analytics. Lecture notes in computer science, vol 11190. Springer, Cham, pp 1-23. 10.1007/978-3-030-01388-2_1

[28] Chandler T, Cordeil M, Czauderna T, Dwyer T, Glowacki J, Goncu C et al (2015) Immersive analytics. In: Proceedings of 2015 big data visual analytics, IEEE, Hobart, 22-25 September 2015. 10.1109/BDVA.2015.7314296

[29] Kraus M, Klein K, Fuchs J, Keim DA, Schreiber F, Sedlmair M (2021) The value of immersive visualization. IEEE Comput Graph Appl 41(4):125-132. 10.1109/MCG.2021.3075258 10.1109/MCG.2021.3075258 34264822

[30] Kraus M, Fuchs J, Sommer B, Klein K, Engelke U, Keim D et al (2022) Immersive analytics with abstract 3D visualizations: a survey. Comput Graph Forum 41(1):201-229. 10.1111/cgf.14430

[31] Spittle B, Frutos-Pascual M, C, Williams I (2022) A review of interaction techniques for immersive environments. IEEE Trans Vis Comput Graph Creed. 10.1109/TVCG.2022.3174805 10.1109/TVCG.2022.3174805 35552136

[32] Büschel W, Chen J, Dachselt R, Drucker S, Dwyer T, Görg C et al (2018) Interaction for immersive analytics. In: Marriott K, Schreiber F, Dwyer T, Klein K, Riche NH, Itoh T et al (eds) Immersive analytics. Lecture notes in computer science, vol 11190. Springer, Cham, pp 95-138. 10.1007/978-3-030-01388-2_4

[33] Jansen Y, Dragicevic P, Isenberg P, Alexander J, Karnik A, Kildal J et al (2015) Opportunities and challenges for data physicalization. In: Proceedings of the 33rd annual ACM conference on human factors in computing systems, Association for Computing Machinery, Seoul, 18-23 April 2015. 10.1145/2702123.2702180

[34] Ens B, Bach B, Cordeil M, Engelke U, Serrano M, Willett W et al (2021) Grand challenges in immersive analytics. In: Proceedings of the 2021 CHI conference on human factors in computing systems, Association for Computing Machinery, Yokohama, 8-13 May 2021. 10.1145/3411764.3446866

[35] Fonnet A, Prié Y (2021) Survey of immersive analytics. IEEE Trans Vis Comput Graph 27(3):2101-2122. 10.1109/TVCG.2019.2929033 10.1109/TVCG.2019.2929033 31352344

[36] Marriott K, Chen J, Hlawatsch M, Itoh T, Nacenta MA, Reina G, Stuerzlinger W (2018) Immersive analytics: time to reconsider the value of 3D for information visualisation. In: Marriott K, Schreiber F, Dwyer T, Klein K, Riche NH, Itoh T et al (eds) Immersive analytics. Lecture notes in computer science, vol 11190. Springer, Cham, pp 25-55. 10.1007/978-3-030-01388-2_2

[37] Hertel J, Karaosmanoglu S, Schmidt S, Bräker J, Semmann M, Steinicke F (2021) A taxonomy of interaction techniques for immersive augmented reality based on an iterative literature review. In: Proceedings of 2021 IEEE international symposium on mixed and augmented reality, IEEE, Bari, 4-8 October 2021. 10.1109/ISMAR52148.2021.00060

[38] Harrington MCR, Bledsoe Z, Jones C, Miller J, Pring T (2021) Designing a virtual arboretum as an immersive, multimodal, interactive, data visualization virtual field trip. Multimodal Technol Interact 5(4):18. 10.3390/mti5040018

[39] Hubenschmid S, Wieland J, Fink DI, Batch A, Zagermann J, Elmqvist N et al (2022) ReLive: bridging in-situ and ex-situ visual analytics for analyzing mixed reality user studies. In: Proceedings of the 2022 CHI conference on human factors in computing systems, ACM, New Orleans, 29 April-5 May 2022. 10.1145/3491102.3517550

[40] Jetter HC, Schröder JH, Gugenheimer J, Billinghurst M, Anthes C, Khamis M et al (2021) Transitional interfaces in mixed and cross-reality: a new frontier? In: Proceedings of the 2021 conference on interactive surfaces and spaces, Association for Computing Machinery, Lodz, 14-17 November 2021. 10.1145/3447932.3487940

[41] Kotlarek J, Kwon OH, Ma KL, Eades P, Kerren A, Klein K et al (2020) A study of mental maps in immersive network visualization. In: Proceedings of 2020 IEEE pacific visualization symposium (PacificVis), IEEE, Tianjin, 3-5 June 2020. 10.1109/PacificVis48177.2020.4722

[42] Billinghurst M, Cordeil M, Bezerianos A, Margolis T (2018) Collaborative immersive analytics. In: Marriott K, Schreiber F, Dwyer T, Klein K, Riche NH, Itoh T et al (eds) Immersive analytics. Lecture notes in computer science, vol 11190. Springer, Cham, pp 221-257. 10.1007/978-3-030-01388-2_8

[43] Chandler T, Morgan T, Kuhlen TW (2018) Exploring immersive analytics for built environments. In: Marriott K, Schreiber F, Dwyer T, Klein K, Riche NH, Itoh T et al (eds) Immersive analytics. Lecture notes in computer science, vol 11190. Springer, Cham, pp 331-357. 10.1007/978-3-030-01388-2_11

[44] Czauderna T, Haga J, Kim J, Klapperstück M, Klein K, Kuhlen T et al (2018) Immersive analytics applications in life and health sciences. In: Marriott K, Schreiber F, Dwyer T, Klein K, Riche NH, Itoh T et al (eds) Immersive analytics. Lecture notes in computer science, vol 11190. Springer, Cham, pp 289-330. 10.1007/978-3-030-01388-2_10

[45] Masrur A, Zhao J, Wallgrün JO, LaFemina P, Klippel A (2017) Immersive applications for informal and interactive learning for earth science. In: Proceedings of workshop on immersive analytics: Exploring future interaction and visualization technologies for data analytics. In conjunction with IEEE VIS, IEEE, Phoenix, 1 October 2017.

[46] Norrby M, Grebner C, Eriksson J, Boström J (2015) Molecular rift: virtual reality for drug designers. J Chem Inf Model 55(11):2475-2484. 10.1021/acs.jcim.5b00544 10.1021/acs.jcim.5b00544 26558887

[47] Bienroth D, Nim HT, Garkov D, Klein K, Jaeger-Honz S, Ramialison M et al (2022) Spatially resolved transcriptomics in immersive environments. Vis Comput Ind Biomed Art 5(1):2. 10.1186/s42492-021-00098-6 10.1186/s42492-021-00098-6 PMC8743310 35001220

[48] Yonker SB, Korshak OO, Hedstrom T, Wu A, Atre S, Schulze JP (2019) 3D medical image segmentation in virtual reality. Electronic Imaging. arXiv preprint arXiv:2103.10504

[49] Radianti J, Majchrzak TA, Fromm J, Wohlgenannt I (2020) A systematic review of immersive virtual reality applications for higher education: design elements, lessons learned, and research agenda. Comput Educ 147:103778. 10.1016/j.compedu.2019.103778

[50] Thomas BH, Welch GF, Dragicevic P, Elmqvist N, Irani P, Jansen Y, Schmalstieg D, Tabard A, ElSayed NAM, Smith RT, Willett W (2018) Situated Analytics, Springer International Publishing, Cham, pp 185–220. 10.1007/978-3-030-01388-2_7

[51] Willett W, Jansen Y, Dragicevic P (2017) Embedded data representations. IEEE Trans Vis Comput Graph 23(1):461-470. 10.1109/TVCG.2016.2598608 10.1109/TVCG.2016.2598608 27875162

[52] Scharf AK, Belant JL, Beyer DE Jr, Wikelski M, Safi K (2018) Habitat suitability does not capture the essence of animal-defined corridors. Mov Ecol 6:18. 10.1186/s40462-018-0136-2 10.1186/s40462-018-0136-2 PMC6158861 30275955

[53] Schlägel UE, Grimm V, Blaum N, Colangeli P, Dammhahn M, Eccard JA et al (2020) Movement-mediated community assembly and coexistence. Biol Rev 95(4):1073-1096. 10.1111/brv.12600 10.1111/brv.12600 32627362

[54] Edelhoff H, Signer J, Balkenhol N (2016) Path segmentation for beginners: an overview of current methods for detecting changes in animal movement patterns. Mov Ecol 4(1):21. 10.1186/s40462-016-0086-5 10.1186/s40462-016-0086-5 PMC5010771 27595001

[55] Demšar U, Buchin K, Cagnacci F, Safi K, Speckmann B, Van de Weghe N et al (2015) Analysis and visualisation of movement: an interdisciplinary review. Mov Ecol 3:5. 10.1186/s40462-015-0032-y 10.1186/s40462-015-0032-y PMC4395897 25874114

[56] Houle A, Wrangham RW (2021) Contest competition for fruit and space among wild chimpanzees in relation to the vertical stratification of metabolizable energy. Anim Behav 175:231-246. 10.1016/j.anbehav.2021.03.003

[57] Smith JE, Pinter-Wollman N (2021) Observing the unwatchable: integrating automated sensing, naturalistic observations and animal social network analysis in the age of big data. J Anim Ecol 90(1):62-75. 10.1111/1365-2656.13362 10.1111/1365-2656.13362 33020914

[58] Merkle JA, Sigaud M, Fortin D (2015) To follow or not? How animals in fusion-fission societies handle conflicting information during group decision-making. Ecol Lett 18(8):799-806. 10.1111/ele.12457 10.1111/ele.12457 26013202

[59] Harel R, Alavi S, Ashbury AM, Aurisano J, Berger-Wolf T, Davis GH et al (2022) Life in 2.5D: animal movement in the trees. Front Ecol Evol 10:801850. 10.3389/fevo.2022.801850

[60] van Wijk RE, Kölzsch A, Kruckenberg H, Ebbinge BS, Müskens GJDM, Nolet BA (2012) Individually tracked geese follow peaks of temperature acceleration during spring migration. Oikos 121(5):655-664. 10.1111/j.1600-0706.2011.20083.x

[61] Jolles JW, King AJ, Killen SS (2020) The role of individual heterogeneity in collective animal behaviour. Trends Ecol Evol 35(3):278-291. 10.1016/j.tree.2019.11.001 10.1016/j.tree.2019.11.001 31879039

[62] Shettleworth SJ (2009) The evolution of comparative cognition: is the snark still a boojum? Behav Processes 80(3):210-217. 10.1016/j.beproc.2008.09.001 10.1016/j.beproc.2008.09.001 18824222

[63] Browning E, Bolton M, Owen E, Shoji A, Guilford T, Freeman R (2018) Predicting animal behaviour using deep learning: GPS data alone accurately predict diving in seabirds. Methods Ecol Evol 9(3):681-692. 10.1111/2041-210X.12926

[64] Lele SR, Merrill EH, Keim J, Boyce MS (2013) Selection, use, choice and occupancy: clarifying concepts in resource selection studies. J Anim Ecol 82(6):1183-1191. 10.1111/1365-2656.12141 10.1111/1365-2656.12141 24499379

[65] Demšar U, Long JA, Benitez-Paez F, Brum Bastos V, Marion S, Martin G et al (2021) Establishing the integrated science of movement: bringing together concepts and methods from animal and human movement analysis. Int J Geogr Inf Sci 35(7):1273-1308. 10.1080/13658816.2021.1880589

[66] Zhang Y (2020) Towards 641 visual exploration and analysis of environmental features for animal behaviour studies. Dissertation, University of Konstanz

[67] Loke LHL, Chisholm RA (2022) Measuring habitat complexity and spatial heterogeneity in ecology. Ecol Lett 25(10):2269-2288. 10.1111/ele.14084 10.1111/ele.14084 PMC9804605 35977844

[68] Picardi S, Coates P, Kolar J, O'Neil S, Mathews S, Dahlgren D (2022) Behavioural state-dependent habitat selection and implications for animal translocations. J Appl Ecol 59(2):624-635. 10.1111/1365-2664.14080

[69] Lochhead I, Hedley N, Çöltekin A, Fisher B (2022) The immersive mental rotations test: evaluating spatial ability in virtual reality. Front Virtual Real 3:820237. 10.3389/frvir.2022.820237

[70] Nathan R, Monk CT, Arlinghaus R, Adam T, Alós J, Assaf M et al (2022) Big-data approaches lead to an increased understanding of the ecology of animal movement. Science 375(6582):eabg1780. 10.1126/science.abg1780 10.1126/science.abg1780 35175823

[71] Zhang Y, Klein K, Deussen O, Gutschlag T, Storandt S (2022) Robust visualization of trajectory data. Inf Technol 64(4-5):181-191. 10.1515/itit-2022-0036

[72] Yen JDL, Tonkin Z, Lyon J, Koster W, Kitchingman A, Stamation K et al (2019) Integrating multiple data types to connect ecological theory and data among levels. Front Ecol Evol 7:95. 10.3389/fevo.2019.00095

[73] Wikelski M, Mueller U, Scocco P, Catorci A, Desinov LV, Belyaev MY et al (2020) Potential short-term earthquake forecasting by farm animal monitoring. Ethology 126(9):931-941. 10.1111/eth.13078

[74] Hao ZZ, Wang C, Sun ZK, Zhao DX, Sun BQ, Wang HJ et al (2021) Vegetation structure and temporality influence the dominance, diversity, and composition of forest acoustic communities. For Ecol Manage 482:118871. 10.1016/j.foreco.2020.118871

[75] Odom KJ, Araya-Salas M, Morano JL, Ligon RA, Leighton GM, Taff CC et al (2021) Comparative bioacoustics: a roadmap for quantifying and comparing animal sounds across diverse taxa. Biol Rev 96(4):1135-1159. 10.1111/brv.12695 10.1111/brv.12695 33652499

[76] Sawyer A, Gleeson A (2018) Animal models and virtual reality. BioTechniques 65(2):55-60. 10.2144/btn-2018-0104 10.2144/btn-2018-0104 30091390

[77] Oxley JA, Santa K, Meyer G, Westgarth C (2022) A systematic scoping review of human-dog interactions in virtual and augmented reality: the use of virtual dog models and immersive equipment. Front Virtual Real 3:782023. 10.3389/frvir.2022.782023

[78] Thurley K, Ayaz A (2017) Virtual reality systems for rodents. Curr Zool 63(1):109-119. 10.1093/cz/zow070 10.1093/cz/zow070 PMC5804145 29491968

[79] Taube JS, Valerio S, Yoder RM (2013) Is navigation in virtual reality with fMRI really navigation? J Cogn Neurosci 25(7):1008-1019. 10.1162/jocn_a_00386 10.1162/jocn_a_00386 PMC10271117 23489142

[80] Stowers JR, Hofbauer M, Bastien R, Griessner J, Higgins P, Farooqui S et al (2017) Virtual reality for freely moving animals. Nat Methods 14(10):995-1002. 10.1038/nmeth.4399 10.1038/nmeth.4399 PMC6485657 28825703

[81] Yang YL, Jenny B, Dwyer T, Marriott K, Chen HH, Cordeil M (2018) Maps and globes in virtual reality. Comput Graph Forum 37(3):427-438. 10.1111/cgf.13431

[82] Cechova M (2020) Ten simple rules for biologists initiating a collaboration with computer scientists. PLoS Comput Biol 16(10):e1008281. 10.1371/journal.pcbi.1008281 10.1371/journal.pcbi.1008281 PMC7580977 33091012

[83] Leek JT, Peng RD (2015) Reproducible research can still be wrong: adopting a prevention approach. Proc Natl Acad Sci USA 112(6):1645-1646. 10.1073/pnas.1421412111 10.1073/pnas.1421412111 PMC4330755 25670866

[84] Kölzsch A, Davidson SC, Kays R, Lang I, Lohr A, Scharf A et al (2021) MoveApps: platform to share and use movement data analysis tools. https://www.moveapps.org/. Accessed 17 May 2023

[85] Demšar J, Curk T, Erjavec A, Gorup Č, Hočevar T, Milutinovič M et al (2013) Orange: data mining toolbox in python. J Mach Learn Res 14:2349-2353

[86] Hucka M, Nickerson DP, Bader GD, Bergmann FT, Cooper J, Demir E et al (2015) Promoting coordinated development of community-based information standards for modeling in biology: the COMBINE initiative. Front Bioeng Biotechnol 3:19. 10.3389/fbioe.2015.00019 10.3389/fbioe.2015.00019 PMC4338824 25759811

[87] Waltemath D, Golebiewski M, Blinov ML, Gleeson P, Hermjakob H, Hucka M et al (2020) The first 10 years of the international coordination network for standards in systems and synthetic biology (COMBINE). J Integr Bioinform 17(2-3):20200005. 10.1515/jib-2020-0005 10.1515/jib-2020-0005 PMC7756615 32598315

[88] Hahne F, Huber W, Gentleman R, Falcon S (2008) Bioconductor case studies. Springer, New York. 10.1007/978-0-387-77240-0

[89] Pirolli P, Card S (2005) The sensemaking process and leverage points for analyst technology as identified through cognitive task analysis. In: Proceedings of international conference on intelligence analysis, the Office of the Assistant Director of Central Intelligence for Analysis and Production, McLean, 2-4 May 2005.

[90] Cagnacci F, Boitani L, Powell RA, Boyce MS (2010) Animal ecology meets GPS-based radiotelemetry: a perfect storm of opportunities and challenges. Philos Trans R Soc Lond B Biol Sci 365(1550):2157-2162. 10.1098/rstb.2010.0107 10.1098/rstb.2010.0107 PMC2894970 20566493

[91] Urbano F, Cagnacci F, Calenge C, Dettki H, Cameron A, Neteler M (2010) Wildlife tracking data management: a new vision. Philos Trans R Soc Lond B Biol Sci 365(1550):2177-2185. 10.1098/rstb.2010.0081 10.1098/rstb.2010.0081 PMC2894960 20566495

[92] Helbig C, Bauer HS, Rink K, Wulfmeyer V, Frank M, Kolditz O (2014) Concept and workflow for 3D visualization of atmospheric data in a virtual reality environment for analytical approaches. Environ Earth Sci 72(10):3767-3780. 10.1007/s12665-014-3136-6

[93] Sacha D, Stoffel A, Stoffel F, Kwon BC, Ellis G, Keim DA (2014) Knowledge generation model for visual analytics. IEEE Trans Vis Comput Graph 20(12):1604-1613. 10.1109/TVCG.2014.2346481 10.1109/TVCG.2014.2346481 26356874

[94] Börner K, Bueckle A, Ginda M (2019) Data visualization literacy: definitions, conceptual frameworks, exercises, and assessments. Proc Natl Acad Sci USA 116(6):1857-1864. 10.1073/pnas.1807180116 10.1073/pnas.1807180116 PMC6369751 30718386

[95] Vermeulen N, Parker JN, Penders B (2013) Understanding life together: A brief history of collaboration in biology. Endeavour 37(3):162–171, 10.1016/j.endeavour.2013.03.001 10.1016/j.endeavour.2013.03.001 PMC3878597 23578694

